# Using Eye Aspect Ratio to Enhance Fast and Objective Assessment of Facial Paralysis

**DOI:** 10.1155/2020/1038906

**Published:** 2020-01-29

**Authors:** Jialing Feng, Zhexiao Guo, Jun Wang, Guo Dan

**Affiliations:** ^1^School of Biomedical Engineering, Health Science Center, Shenzhen University, Shenzhen, China; ^2^Shenzhen Institute of Neuroscience, Shenzhen, China; ^3^Shenzhen Hospital, Southern Medical University, Shenzhen, China

## Abstract

A rapid and objective assessment of the severity of facial paralysis allows rehabilitation physicians to choose the optimal rehabilitation treatment regimen for their patients. In this study, patients with facial paralysis were enrolled as study objects, and the eye aspect ratio (EAR) index was proposed for the eye region. The correlation between EAR and the facial nerve grading system 2.0 (FNGS 2.0) score was analyzed to verify the ability of EAR to enhance FNGS 2.0 for the rapid and objective assessment of the severity of the facial paralysis. Firstly, in order to accurately calculate the EAR, we constructed a landmark detection model based on the face images of facial paralysis patients (FP-FLDM). Evaluation results showed that the error rate of facial feature point detection in patients with facial paralysis of FP-FLDM is 17.1%, which was significantly superior to the landmark detection model based on normal face images (NF-FLDM). Secondly, in this study, the Fréchet distance was used to calculate the difference in bilateral EAR of facial paralysis patients and to verify the correlation between this difference and the corresponding FNGS 2.0 score. The results showed that the higher the FNGS 2.0 score , the greater the difference in bilateral EAR. The correlation coefficient between the bilateral EAR difference and the corresponding FNGS 2.0 score was 0.9673, indicating a high correlation. Finally, through a 10-fold crossvalidation, we can know that the accuracy of scoring the eyes of patients with facial paralysis using EAR was 85.7%, which can be used to enhance the objective and rapid assessment of the severity of facial paralysis by FNGS 2.0.

## 1. Introduction

Facial paralysis can lead to the loss of autonomic motor function of the unilateral mimetic muscles of the face. Accurate and objective evaluation of the degree of facial nerve motor function damage is key for the treatment and rehabilitation of facial paralysis patients. Clinically, the House–Brackmann grading system (HBGS) [1] is used to evaluate and grade facial nerve motor dysfunction in patients with facial paralysis. Because the description of symptoms of adjacent facial paralysis grades in HBGS is ambiguous, the evaluation is prone to subjective differences. Moreover, HBGS cannot reflect motor dysfunction in the local regions of the eyebrow, eye, mouth, and nose, which affects the design of follow-up treatment and rehabilitation regimen for facial paralysis patients. Therefore, in 2009, the American Academy of Otorhinolaryngology-Head and Neck Surgery Facial Neuropathy proposed the facial nerve grading scale 2.0 (FNGS 2.0) [2]. FNGS 2.0 assesses the severity of facial paralysis by manually measuring facial features in images, which makes it difficult to rapidly and objectively determine the severity of facial paralysis. The development of an objective and quantitative automated method to rapidly assess the severity of facial paralysis is extremely important for the design of subsequent rehabilitation regimens.

To objectively assess the severity of facial paralysis, Tomat and Manktelow [3] developed a measurement system to assess the asymmetry of local facial regions targeted at the displacement and angle changes of the landmarks of the still mouth and smiling mouth in 2005. In 2010, Liu et al. [4] captured images of facial paralysis patients with different facial expressions, compared the shape of the region and area change of the eye, forehead, nose, and mouth, and calculated these changes to evaluate the degree of facial nerve injury. In 2016, Wang et al. [5] proposed a facial symmetry quantitative evaluation method based on the principle of local mirror symmetry. The method objectively and quantitatively assesses the severity of facial paralysis through the 3 steps of local region localization, extraction of asymmetric features, and quantification of asymmetry of the bilateral face. All three of them quantified the features of local facial regions to evaluate the symmetry of facial structures. However, Tomat's work measured the symmetry of the mouth of normal people without considering other local facial areas. Liu's work could not achieve real-time evaluation; Wang's work was not applied to studies related to facial paralysis. Therefore, the present study refers to the research ideas of Wang et al. to propose quantitative indicators for local regions of the face in real time to objectively and quantitatively assess the severity of the corresponding regions of facial paralysis. How to accurately obtain 2 d or 3 d facial information of patients with facial paralysis and evaluate the severity of facial paralysis based on this characteristic information is the main challenge. Based on the coordinates of feature points to extract facial information, the accurate detection of feature points and quantitative evaluation of facial structure and motor function symmetry are the main problems to be solved. Therefore, this study trained a new feature point detection model to improve the accuracy of feature point detection and proposed a regional index to quantify the motion symmetry of facial regions.

**Figure 1:**
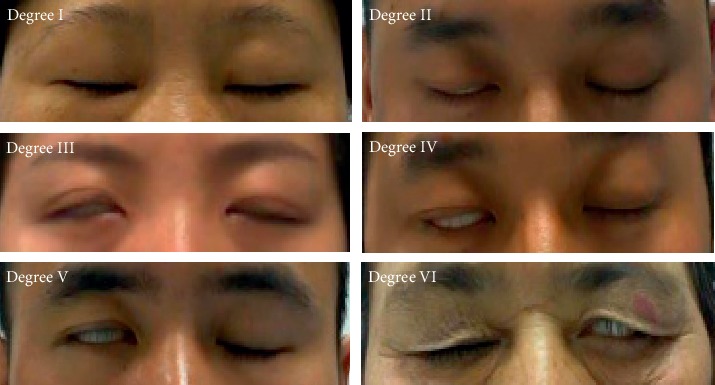
Eye region graded by severity in FNGS 2.0.

In FNGS 2.0, facial paralysis patients need to complete the facial expressions of the corresponding regions for evaluation. The corresponding regions include the eyebrows, eyes, nose, and mouth, and common facial expressions include opening eyes, closing eyes, baring teeth, and bulging mouth. The movements of the eyes and mouth better manifest the asymmetry of facial expressions caused by facial nerve injury of the affected side in facial paralysis patients than those of the eyebrows and nose, and the amplitude of movements on both sides of the eye is easier to observe and compare than those in the mouth region (Figure 1). In addition to damaging the facial features, facial paralysis also has a great impact on the patient's eyes. Facial paralysis may cause enlarged ocular fissures and an inability to close the eyes or blink. In particular, prolonged exposure of the cornea during sleep can cause dryness of the cornea, which can easily lead to eye infection and damaged vision in severe cases [6]. Studies of the objective and rapid assessment of eye motor function of facial paralysis patients are needed to develop optimal treatment regimens for facial paralysis patients to help them recover as quickly as possible. Therefore, this study proposes the eye aspect ratio (EAR) index, which targets the eye region of facial paralysis patients and experimentally analyzes the feasibility of applying the difference in bilateral EAR in the objective and rapid assessment of the severity of facial paralysis.

**Figure 2:**
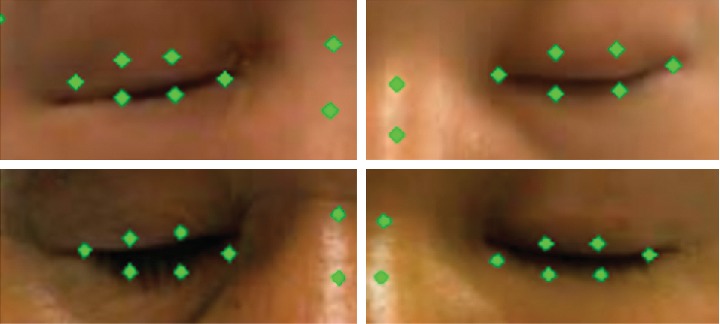
Detection results of closed-eye images in patients with facial paralysis by the landmark detection model based on normal face images: (a) Left eye (b) Right eye.

Common facial landmark detection models are constructed based on normal facial databases, such as 300-W [7] and AFLW [8], and are thus not suitable for face images of facial paralysis patients. The detection results of such models for closed-eye images are shown in Figure 2. Facial paralysis patients exhibit strange facial expressions due to facial nerve injury and cannot open and close eyes, bare teeth and bulge mouth normally on the affected side. Therefore, existing facial landmark detection models based on normal face images are not suitable for the calculation of EAR.

**Figure 3:**
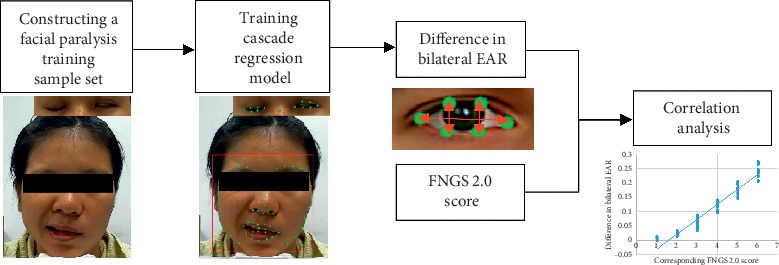
Study design and flowchart.

As shown in Figure 3, in this study, to verify the feasibility of EAR in enhancing FNGS 2.0 to rapidly and objectively assess the eye motor function of facial paralysis patients, we first constructed a dataset of facial landmark images of facial paralysis patients. A facial landmark detection model for facial paralysis patients (FP-FLDM) was constructed using this dataset. The landmark detection model was used to acquire information on eye landmarks in the movement image series to calculate the EAR index during the eye movement process of facial paralysis patients. The Fréchet distance of the EAR index curve was calculated to represent the difference in bilateral EAR. Finally, the correlation between the difference in bilateral EAR and the corresponding FNGS 2.0 score was analyzed by experiments to prove the feasibility of EAR in enhancing FNGS 2.0 to objectively and rapidly assess eye motor function.

## 2. Methods

### 2.1. Facial Nerve Grading Scale 2.0(FNGS2.0)

To address the inability of HBGS to reflect local motor dysfunction of the eyebrow, eye, mouth, and nose and the overlapping and inaccurate evaluation rules of adjacent facial paralysis grades, the American Academy of Otorhinolaryngology-Head and Neck Surgery Facial Neuropathy proposed FNGS 2.0 in 2009. FNGS 2.0 can assess changes in the eyebrow, eye, nose, and mouth regions on a scale of 1–6 according to the degree of motor dysfunction and can evaluate the severity of complications of facial paralysis on a scale of 0–3. The sum of these two scores is used to evaluate the severity of facial paralysis. The specific scoring rules of FNGS 2.0 are shown in Table 1.

### 2.2. Facial Landmark Detection Model

**Figure 4:**
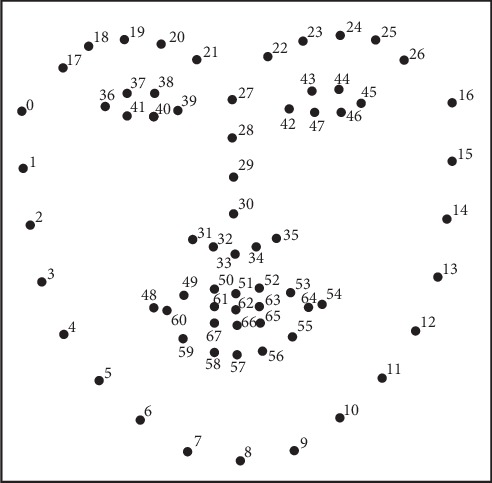
The 68 points mark-up used for actual landmark distribution [7].

The cascaded regression model is a mapping function that learns directly from the facial appearance to the facial shape (or the parameters of the facial shape model) and then establishes the correspondence from the appearance to the shape. This method does not require complex facial shapes, and appearances for modeling is simple and efficient and achieves good positioning effects in controllable scenarios (human faces collected under laboratory conditions) and noncontrollable scenarios (face images from the Internet) [9]. The basis of the tree algorithm is the decision tree. The decision tree is widely used in statistics, data mining, and machine learning because it is easy to understand, easy to construct, and rapid [10]. Therefore, this study employed the ensemble of regression tree (ERT) algorithm, a regression tree method based on gradient boosting learning. The residual regression tree (gradient boosting decision tree, GBDT) algorithm is used to construct each level of regressors, where each regressor is composed of multiple decision trees, and the parameters of each decision tree are obtained according to the residual between the current landmark distribution and the actual landmark distribution and through training of randomly selected pixel pairs. The actual landmark distribution in this paper is shown in Figure 4.

The construction process of the model mainly includes 3 main modules: generating the training sample set, training the optimal weak regressor, and updating the training sample set. The marked face images of facial paralysis patients were used as a training set, and the initial positions of the facial landmarks in each face image were randomly generated as the training sample T. The GBDT algorithm was used to reduce the normalized mean error (NME) between the current landmark distribution coordinates and the actual landmark distribution coordinates [11], the least squares method was used to minimize errors, the cascade regression factor of each level was obtained, and the optimal weak regressor was obtained through continuous training. The NME can be written as follows:

(1) $$\begin{array}{c} \text{NME}=\frac{1}{N}\overset{N}{\underset{i=1}{\sum }}\frac{{\left\|z_{i}-s_{i}\right\|}^{2}}{d_{i}}, \end{array}$$

where *z*_*i*_ denotes the current landmark distribution coordinates of the landmarks of the face, *s*_*i*_ is the actual landmark distribution coordinates and *d*_*i*_ is the interocular distance.

The core formula is shown below, where ${\hat{S}}^{\left(t\right)}$ represents the shape of the *t*-th regressor and *r*_*t*_ represents the update of the *t*-th regressor.

(2) $$\begin{array}{c} {\hat{S}}^{\left(t+1\right)}={\hat{S}}^{\left(t\right)}+r_{t}\left(I,{\hat{S}}^{\left(t\right)}\right). \end{array}$$

The detection process of the model was to first initialize the input eye landmarks, extract eye features according to the current landmark distribution, input the extracted features into the established weak regressor, and update the current landmark distribution according to the residuals of the landmark distribution, which contains the eye landmark information required for calculating the EAR index.

### 2.3. Calculation of the Difference in Bilateral EAR

EAR refers to the aspect ratio of the eye region, which is often used to calculate the temporal consistency and speed of left and right eye blinks [12] and in fatigue detection [11,13–15]. There has been no report of the application of EAR to the objective assessment of facial paralysis. In this study, EAR was used to characterize the displacement changes of the eye landmarks of facial paralysis patients in the movement image series, and then the difference in bilateral eye movements of facial paralysis patients was evaluated according to the changes in EAR.

In both normal face images and facial paralysis face images, the landmarks of the nasal root and the nose tip on the face changed little during the movements (displacement changes of less than 2 pixels). To increase the difference in bilateral eye movements to improve the performance of the difference in EAR characteristics and based on the observation that the movements of the bilateral eyebrows were also inconsistent during the process of eye opening and closing in facial paralysis patients, the Euclidean distance from the centre of the eyebrows to the tip of the nose divided by the length of the nose was used as an amplification factor for EAR [16]. The formula for calculating the one-side EAR is as follows: in the t-frame image, the EAR of the left eye is EAR_Lt_, the EAR of the right eye is EAR_Rt_, and LM_n_ is the landmark corresponding to the index *n*:

(3) $$\begin{array}{c} {\text{EAR}}_{\text{Lt}}=\frac{\left\|{\text{LM}}_{37}-{\text{LM}}_{41}\right\|+\left\|{\text{LM}}_{38}-{\text{LM}}_{40}\right\|}{2\;*\;\left\|{\text{LM}}_{36}-{\text{LM}}_{39}\right\|}*\frac{\left\|{\text{LM}}_{19}-{\text{LM}}_{33}\right\|}{\left\|{\text{LM}}_{27}-{\text{LM}}_{33}\right\|},\\ {\text{EAR}}_{\text{Rt}}=\frac{\left\|{\text{LM}}_{43}-{\text{LM}}_{47}\right\|+\left\|{\text{LM}}_{44}-{\text{LM}}_{46}\right\|}{2\;*\;\left\|{\text{LM}}_{42}-{\text{LM}}_{45}\right\|}*\frac{\left\|{\text{LM}}_{24}-{\text{LM}}_{33}\right\|}{\left\|{\text{LM}}_{27}-{\text{LM}}_{33}\right\|}. \end{array}$$

**Figure 5:**
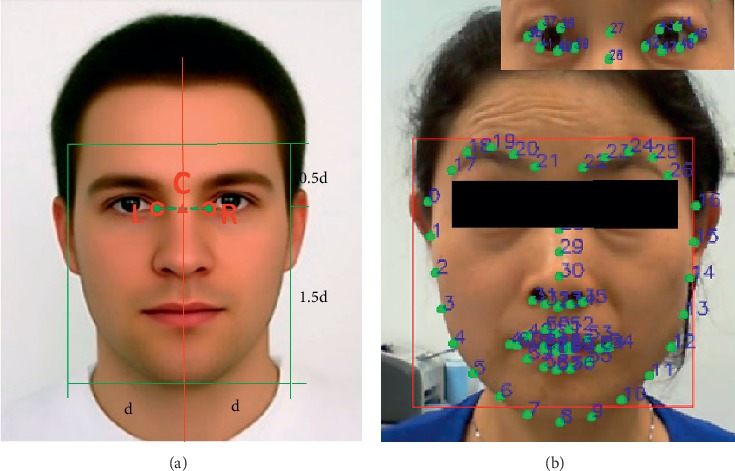
(a) Normalized geometric model of the face; (b) landmark detection model index numbers.

In addition, when facial paralysis patients perform facial expressions, their heads often swing. Direct calculation of the landmark displacement of each frame in the movement image series will result in a large error, and thus it is necessary to perform tilt correction on the faces in the images. Moreover, because the size of the face area of each subject is different, geometric normalization of the faces is required [17]. According to the coordinate values of the two eye corners, the face was rotated to calibrate the tilt. The distance between the two eyes was set as d, and the midpoint was C. In the rectangular feature area of the face, with point C as the reference, the distance to each side was set as d, and distances of 0.5 d and 1.5 d on the vertical direction were set. The normalized geometric models of the face and facial landmarks are shown in Figure 5.

To establish the relationship between EAR and the FNGS 2.0 score, the Fréchet distance was used to represent the difference in bilateral EAR. In 1994, Eiter and Mannila proposed the definition of the discrete Fréchet distance [18]: by calculating the Euclidean distance between the pair of sequential points of two trajectory curves, the maximum value of the sequence is selected as the spatial similarity of the two curves. The core formula is as follows:

(4) $$\begin{array}{c} {\text{max distance}}_{\left(P,Q\right)}=\max\left[\sqrt{\overset{n}{\underset{i=1}{\sum }}{\left(p_{i}-q_{i}\right)}^{2}}\right], \end{array}$$

where *p*_*i*_ denotes the *i*-th EAR difference *P* on left side and *q*_*i*_ is the *i*-th EAR difference *Q* on right side.

In this study, the Fréchet distance was used to represent the bilateral EAR difference of facial paralysis patients. The larger the Fréchet distance, the smaller the similarity of the EAR, that is, the greater the difference in the movements of the bilateral eye regions.

## 3. Experiments

### 3.1. Data and the Construction of the Facial Landmark Detection Model (FLDM)

**Figure 6:**
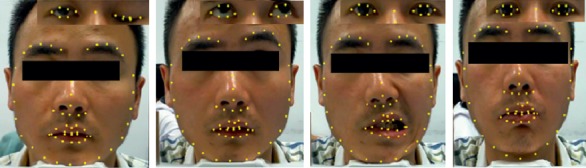
Marked data sets for closed eye, open eye, baring teeth, and bulging mouth from left to right.

The facial image data of facial paralysis patients in this study were derived from the literature [19]. Using FNGS 2.0, rehabilitation doctors of the collaborating hospitals objectively evaluated the eye motor function of 105 patients with facial paralysis with scores of 1–6 corresponding from normal to severe based on the severity of paralyzed eyes: 5 patients received scores of 1 point, 21 patients 2 points, 31 patients 3 points, 13 patients 4 points, 23 patients 5 points, and 12 patients 6 points. The major facial muscles involved in facial paralysis are the occipital frontal muscle, frontal abdomen, orbicularis oculi muscle, zygomatic muscle, oral muscle, and orbicularis oculi muscle. Opening the eyebrows, closing the eyes, baring the teeth, and bulging the mouth help to train these major muscles and help to restore normal motor function of the entire facial expression muscle [20]. As a result, to construct the FLDM, we extracted 4 images each of open eye, closed eye, baring teeth, and bulging mouth from the image series of 105 facial paralysis patients and marked 68 facial landmarks on a total of 420 facial paralysis images. The marked results are shown in Figure 6.

To evaluate the accuracy of the model detection results, the marked data sets were divided into a training set and a testing set containing 336 images and 84 images, respectively. Due to the small number of samples in the training set, to prevent overfitting of the model, data amplification of the training set was necessary. Kazemi and Sullivan [21] trained and tested the cascading regression tree model on the Helen [22] dataset, with 2000 training sets and 330 test sets. We have 336 training data and 84 test data. In order to keep the data volume basically consistent with the reference model, our study amplified the random deformation of the dataset by 10 times. The amplified training set was input into the cascade regressor to perform weak regressor training to generate the model required for subsequent study. We set the decision tree depth parameters as 2, 4, 5, and 10 and the regular term parameters as 0.001, 0.01, 0.05, 0.1, 0.2, 0.3, 0.4, 0.5, 0.6, 0.7, 0.8, and 0.9. Paired combinations of the decision tree depth parameters and regular term parameters were used to construct the FLDM, and finally the model with the highest accuracy was selected for subsequent study.

### 3.2. Evaluation of the FLDM

**Figure 7:**
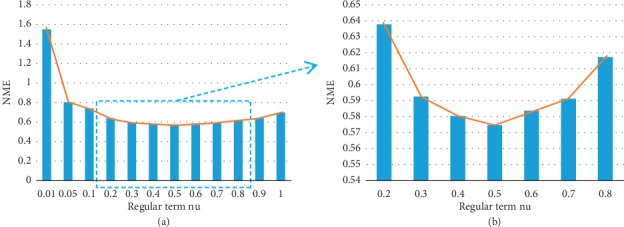
(a) Model error column graph with a tree depth of 5 and regular term parameters from 0.01 to 1.0; (b) model error column graph with a tree depth of 5 and regular term parameters from 0.2 to 0.8.

FLDMs commonly use the NME to evaluate model accuracy [23]. In this study, the NMEs between the model detection values and the standard values of the eye landmark coordinates were calculated as the evaluation standard of the model training effect.  Figure 7(a) shows the model NME output when the tree depth was 5 and the regular term coefficient distribution was adjusted from 0.01 to 1.  Figure 7(b) shows a partial enlargement of Figure 7(a).  Figure 7 shows that the accuracy of the model is highest when the tree depth is 5 and the regular term parameter is 0.5.

**Figure 8:**
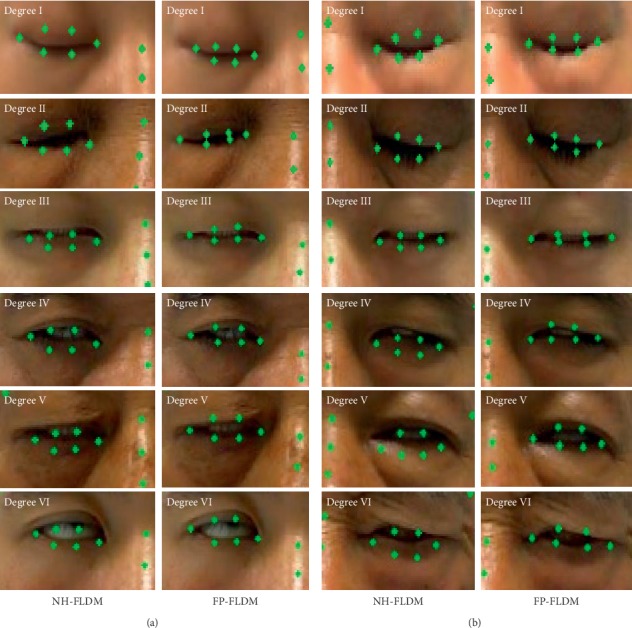
Comparison of NH-FLDM and FP-FLDM in the detection of the closed-eye landmarks of patients with facial paralysis: (a) left eye is the affected side and (b) right eye is the affected side.

**Figure 9:**
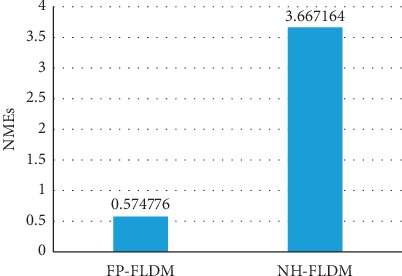
Comparison of average NMEs of NH-FLDM and FP-FLDM in eye detection in the movement image series.

In 2014, Kazemi et al. constructed a cascaded regression tree landmark detection model using normal face data (NH-FLDM). The representative algorithm based on cascading regression is the cascading regression tree model, which is used to learn the mapping function from face appearance to face shape. Our model also uses regression model to predict facial feature points, so it is meaningful to compare with the mainstream regression model. The detection accuracy of NH-FLDM was compared with that of our constructed model (FP-FLDM) on the eye landmarks of facial paralysis patients in the test set.  Figure 8(a) and Figure 8(b) show the results of eye landmark detection of the affected side of facial paralysis using NH-FLDM and FP-FLDM, respectively.  Figure 9 shows the comparison of the NMEs of NH-FLDM and FP-FLDM in the detection of eye landmarks of facial paralysis patients.  Table 2 shows the statistical analysis of the eye landmark detection effects of NH-FLDM and FP-FLDM on movement image series in the test set.

### 3.3. Calculation and Analysis of the Bilateral EAR Difference

**Figure 10:**
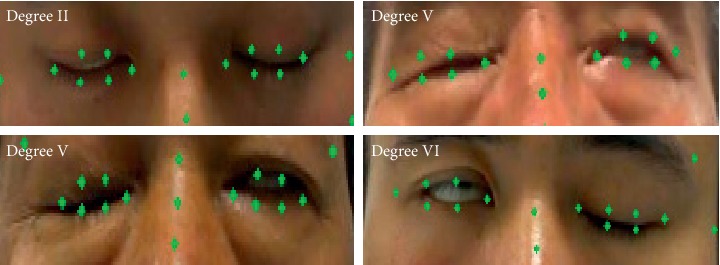
Sample representation of poor detection results of closed eye by FP-FLDM.

**Figure 11:**
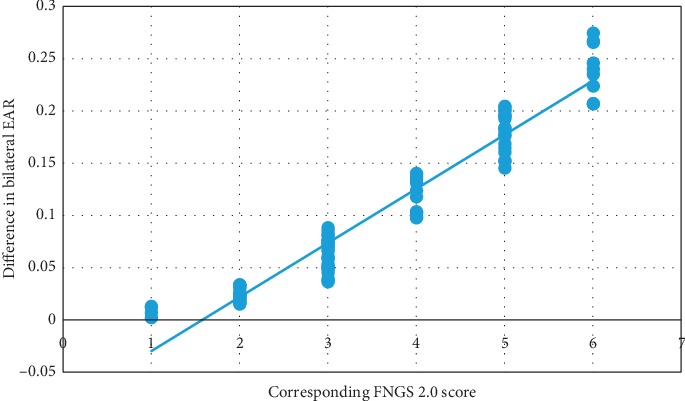
Scatter plot of the difference in bilateral EAR of the 87 samples with the corresponding FNGS 2.0 scores.

**Figure 12:**
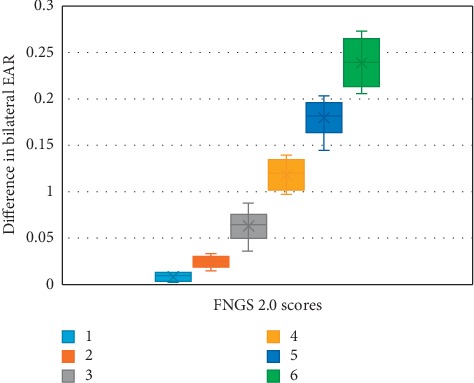
Box plot of the distribution of bilateral EAR differences corresponding to different FNGS 2.0 scores.

**Figure 13:**
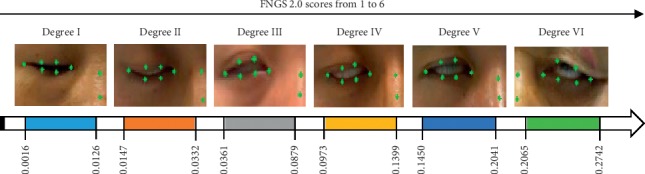
Differences in bilateral EAR and closed-eye images corresponding to different FNGS 2.0 scores.

To analyze the correlation between EAR and the FNGS 2.0 score, we excluded 18 samples incorrectly detected by FP-FLDM. The exclusion criteria are shown in Figure 10.  Table 3 shows the quantity distribution of different FNGS 2.0 scores of the original samples and the remaining 87 samples.  Figure 11 shows the scatter plot and fitted curve of the bilateral EAR difference of the 87 samples versus the corresponding FNGS 2.0 scores.  Figure 12 shows the box plot of the bilateral EAR difference of the 87 samples with the corresponding FNGS2.0 scores.  Figure 13 shows a more intuitive representation of the distribution of the bilateral EAR difference of facial paralysis patients according to different FNGS 2.0 scores.  Table 4 shows the results of correlation analysis between the FNGS 2.0 scores and the corresponding bilateral EAR differences.

## 4. Discussion

To verify the feasibility of EAR in enhancing FNGS 2.0 to rapidly and objectively assess the severity of the corresponding region of facial paralysis, the correlation between the bilateral EAR difference and the FNGS 2.0 score was calculated in the final part of the experiment.  Table 4 shows that the correlation coefficient between the two was 0.9673, indicating that the bilateral EAR difference is highly correlated with the FNGS 2.0 score. As shown in Figures 12 and 13, as the FNGS 2.0 score increases, that is, the degree of eye movement dysfunction becomes more severe, the differences in bilateral EAR gradually increase. The interval of bilateral EAR differences corresponding to different FNGS 2.0 scores was significant, indicating that the bilateral EAR difference can easily distinguish the corresponding FNGS 2.0 score. Moreover, this study was performed on a 3.4 GHz PC based on the Python platform with a processing speed of 30 frames per second, indicating that these indicators can rapidly assess the severity of the corresponding region of facial paralysis. Therefore, in summary, the bilateral EAR difference can objectively and rapidly assess the severity of facial paralysis.

In addition, this study analyzed the evaluation results of 105 cases of facial paralysis with reference to the research method of Lee et al. [24]. The comparison of regional scores and facial paralysis grading showed that both HBGS and FNGS 2.0 had the highest consistency on eye movement function scores and facial paralysis grades, followed by the mouth (Table 5), suggesting that the examination of movement differences in the eye region is meaningful for the objective classification of facial paralysis.

Furthermore, Table 2 shows that in the detection of eye landmarks in the movement image series, the incorrect detection rate of NH-FLDM was 27.6% and the incorrect detection rate of FP-FLDM was 17.1%. In addition, patients with severe facial paralysis have extremely abnormal facial features and are considered difficult samples for landmark detection models. When the NH-FLDM detected the eye landmarks of facial paralysis patients with scores of 5 and 6, the incorrect detection rates were 5.7% and 6.7%, respectively, while those of FP-FLDM were only 4.8% and 1.9%, respectively, indicating a significantly better effect of FP-FLDM compared with NH-FLDM. These results imply that it is essential to construct a facial landmark detection model for facial paralysis patients and that the eye landmark detection model constructed in this study is suitable for patients with facial paralysis.

As an exploratory study, several experiments await completion in the future. Table 5 shows that the severity of the eye region of facial paralysis patients cannot fully represent the final score of the facial paralysis patient. Therefore, we still need to explore the corresponding indicators of the eyebrow, nose, and mouth to enhance FNGS 2.0 scoring as a fast and accurate objective assessment for facial paralysis based on a small number of indicators. In addition, although the performance of FP-FLDM was superior to NH-FLDM in facial paralysis patients, Table 1 shows that 17.1% of patient samples were still not accurately detected. Therefore, the performance of the eye landmark detection model constructed in this paper awaits further improvement.

In this study, a 10-fold cross-validation method was used to randomly divide 87 samples into 10 samples. One percent of the samples was selected for each test, and the other 10% was used to construct the EAR feature dataset. The eigenvalues and FNGS2.0 scores were divided into six categories. The process was repeated 10 times, and the average accuracy was 85.2%.

## 5. Conclusion

In this study, face image data of facial paralysis patients were used as a training set, and a facial landmark detection model for facial paralysis patients was constructed to calculate the EAR. The incorrect detection rate of this model for the eye region of facial paralysis patients was 17.1%, an improvement over currently available detection models. Detection and calculation using the constructed detection model yielded the difference in bilateral EAR of 87 facial paralysis patients. Subsequently, the relationship between the FNGS 2.0 score and the corresponding bilateral EAR difference was analyzed, and the correlation analysis results showed that the two were highly correlated, with a correlation coefficient of 0.9673. By implementing the proposed method and related experiments, this study proves that the bilateral EAR difference can be applied to the objective and rapid assessment of the severity of facial paralysis.

## Figures and Tables

**Table 1:** Facial nerve grading scale 2.0.

Score	Region			
	Brow	Eye	NLF	Oral

1	Normal	Normal	Normal	Normal
2	Slight weakness >75% of normal	Slight weakness >75% of normal, Complete closure with mild effort	Slight weakness >75% of normal	Slight weakness >75% of normal
3	Obvious weakness >50% of normal, Resting symmetry	Obvious weakness >50% of normal, Complete closure with maximal effort	Obvious weakness >50% of normal, Resting symmetry	Obvious weakness >50% of normal, Resting symmetry
4	Asymmetry at rest <50% of normal, Cannot close completely	Asymmetry at rest <50% of normal	Asymmetry at rest <50% of normal	Asymmetry at rest <50% of normal
5	Trace movement	Trace movement	Trace movement	Trace movement
6	No movement	No movement	No movement	No movement

Secondary movement (global assessment)				
Score	Degree of movement			
0	None			
1	Slight synkinesis; minimal contracture			
2	Obvious synkinesis; mild to moderate contracture			
3	Disfiguring synkinesis; severe contracture			

Reporting: sum scores for each region and secondary movement				
Grade	Total score			
I	4			
II	5–9			
III	10–14			
IV	15–19			
V	20–23			
VI	24			

NLF, nasolabial fold.

**Table 2:** Statistics of incorrect detection of eye landmarks in the movement image series by the two models.

FNGS2.0 score	1	2	3	4	5	6	Total
NH-FLDM (%)	1.9	4.8	5.7	2.9	5.7	6.7	27.6
FP-FLDM (%)	0	2.9	5.7	1.9	4.8	1.9	17.1

**Table 3:** Quantity distribution of the original samples and the experimental samples with different FNGS 2.0 scores.

FNGS2.0 score	1	2	3	4	5	6
Original sample	5	21	31	13	23	12
Experimental sample	5	18	25	11	18	10

**Table 4:** Correlation analysis between FNGS 2.0 scores and bilateral EAR differences.

FNGS2.0 score	1	2	3	4	5	6
Median of bilateral EAR difference	0.0071	0.0240	0.0620	0.1186	0.1746	0.2404
Correlation coefficient	0.9673					

**Table 5:** Consistency of facial paralysis grading and HBGS, and FNGS 2.0 scores in different regions (*n* = 105).

	Eyebrow	Eye	Mouth	Nasolabial groove
HBGS	47(44.8%)	53(50.5%)	52(49.5%)	42(40.0%)
FNGS 2.0	52(49.5%)	60(57.1%)	55(52.4%)	43(41.0%)

## Data Availability

The raw/processed data required to reproduce these findings cannot be shared at this time as the data also form part of an ongoing study.
